# Phylogeny and taxonomic synopsis of PoasubgenusPseudopoa (including *Eremopoa* and *Lindbergella*) (Poaceae, Poeae, Poinae)

**DOI:** 10.3897/phytokeys.111.28081

**Published:** 2018-11-14

**Authors:** Lynn J. Gillespie, Robert John Soreng, Evren Cabi, Neda Amiri

**Affiliations:** 1 Research and Collections Division, Canadian Museum of Nature, P.O. Box 3443, Station D, Ottawa, Ontario K1P 6P4, Canada Canadian Museum of Nature Ottawa Canada; 2 Department of Botany, National Museum of Natural History, Smithsonian Institution, Washington, DC 20013-7012, USA University of Ottawa Ottawa Canada; 3 Department of Biology, Faculty of Arts and Sciences, Tekirdağ Namık Kemal University, Tekirdağ, Turkey Smithsonian Institution Washington United States of America; 4 Department of Biology, University of Ottawa, Ottawa, Ontario K1N 6N5, Canada Tekirdağ Namık Kemal University Tekirdağ Turkey

**Keywords:** Annuals, classification, DNA, *
Eremopoa
*, grasses, *
Lindbergella
*, phylogeny, *
Poa
*, Poaceae, taxonomy

## Abstract

*Eremopoa* is a small genus of annual grasses distributed from Egypt to western China. Phylogenetic analyses of plastid and nuclear ribosomal DNA show that *Eremopoa* species, together with the monotypic genus *Lindbergella* and a single species of *Poa* (*P.speluncarum*), are nested within the genus *Poa*, in a clade that we accept as Poasubg.Pseudopoa. Here we accept seven species, four subspecies and four varieties in Poasubg.Pseudopoa. Five new combinations are made: *Poaattalica, P.diaphora* var. *alpina, P.diaphora* var. *songarica, P.nephelochloides* and P.persicasubsp.multiradiata; *P.millii* is proposed as a replacement name for *E.capillaris*; and *Poa* sections *Lindbergella* and *Speluncarae* are proposed. We provide a diagnosis for Poasubg.Pseudopoa, synonymy for and a key to the taxa. Eight lectotypes are designated: *Eragrostisbarbeyi* Post, *Eremopoanephelochloides* Roshev., *Glyceriataurica* Steud., *Nephelochloatripolitana* Boiss. & Blanche, *Poacilicensis* Hance, *Poaparadoxa* Kar. & Kir., Poapersicavar.alpina Boiss and Poapersicasubsp.cypria Sam. *Eremopoamedica* is re-identified as a species of *Puccinellia*.

## Introduction

*Eremopoa* Roshev. is a small, primarily west and central Asian genus of annual grasses. Roshevitz (1934) named the genus *Eremopoa* (Greek: *eremos* = desert, *poa* = fodder / > bluegrass) and included six species of annuals for the former U.S.S.R. Up to that time, one or more of the taxa had been described or treated in *Aira* L. (Trinius 1835), *Eragrostis* Wolf (Post and Autran 1897), *Festuca* L. (Koch 1848), *Glyceria* R. Br. (Fischer and Meyer 1841, Steudel 1854), *Nephelochloa* Boiss. (Grisebach 1852, Boissier and Blanche 1859) and *Poa* L. (Trinius 1830, 1836, Steudel 1854, Boissier 1884, Hackel 1887, Stapf 1897, Ascherson and Graebner 1900). *Poapersica* Trin. is the type species of *Eremopoa*, Festucasect.Pseudopoa K. Koch, Poasubgen.Pseudopoa (K. Koch) Stapf and P.sect.Pseudopoa (K. Koch) Hack. After *Eremopoa* was described, most authors accepted the genus (Grossheim 1939, Köie 1945, Bor 1960, 1968a, 1970, Pavlov and Gamajunova 1964, Tzvelev 1966, 1976, 1989, Scholz 1980, 1981, Tutin 1980, Czerepanov 1981, 1995, Cope 1982, Mill 1985, Clayton and Renvoize 1986, Watson and Dallwitz 1992, Soreng 2003, Valdés and Scholz 2006, Darbyshire 2007, Cabi and Doğan 2012, Nikiforova et al. 2012). Few taxonomists continued to refer the species to *Poa* (Samuelsson 1950, Kovalevskaja 1968). No revision of the genus as a whole exists.

Roshevitz (1934) differentiated the genus *Eremopoa* from *Poa* as: always annuals with long panicle branches arranged in half-whorls; glumes unequal, inferior 1-veined, superior 3-veined; lemmas with obscure keel and lateral veins, apex acuminate or briefly aristate; and callus without lanate hairs. Tzvelev (1976) added the following characteristics: lower glumes 2/7–2/3 the first lemma in length; lemmas somewhat keeled with 5 veins, apex gradually tapering, sometimes with a short cusp, somewhat scabrous due to very short spinules and often pilose in the lower part along the keel and marginal veins; callus obtuse, glabrous or almost glabrous; leaf sheaths closed only at the base and leaf blades flat or loosely folded. The genus is relatively easy to recognise as a set of annuals, whereas *Poa* has few annuals and those are distinct from species included in *Eremopoa*. However, none of the characters by themselves actually differentiates *Eremopoa* from *Poa*. In *Poa*, glumes can also be short, the lower one is commonly 1-veined, the upper one normally 3-veined. Lemmas in *Poa* are usually distinctly keeled, with soft hairs at least on the keel and with an obtuse, acute or acuminate apex. They are rarely weakly keeled (e.g. in sect. Secundae), sometimes glabrous (ca. 15% of spp.) and rarely produce a minute cusp (a cusp occurs more often than acknowledged in the literature, but is usually irregularly expressed). In *Poa*, a dorsal tuft of hairs on the callus is present in 2/3 of the species. In the other species, the callus is sometimes glabrous or has a minute or more developed crown of hairs around the base of the lemma. In addition, *Poa* leaf sheaths are only infrequently closed at the base, most being closed more than 1/10 the length, and leaf blade form runs the gamut from flat and thin to tough and involute. Panicle branches in *Poa* are infrequently whorled with 6 or up to 9 branches per lower node, the normal range is 1 to 5. Although panicle branches are commonly numerous (ranging up to 27) in *Eremopoa*, with most taxa usually having over 5, *E.altaica* (Trin.) Roshev. has 1–5(–7) and *E.songarica* (Schrenk ex Fisch. & C.A. Mey.) Roshev. varies widely with (1–)3–8(–12). *Eremopoa* species are annual with some extreme features usually not found in *Poa*, but, other than abundantly branching panicles, those characteristics are broached in all cases. No one has doubted that *Eremopoa* was closely related to *Poa*.

The taxa placed in *Eremopoa* range from Egypt (Sinai and north coast) across the northern Middle East (Israel, Lebanon, Syria, Iraq, Turkey [Anatolia], Iran), to Afghanistan, Pakistan, northwest India (Himachal Pradesh, Kashmir), western China (Tibet and Xinjiang), north through Transcaucasia into the Caucasus mountains of Russia and across central Asia in Turkmenistan, Uzbekistan, Tajikistan, Kyrgyz Republic and Kazakhstan. Two taxa have been observed elsewhere as waifs: *E.persica* in western Europe (France, Norway) and *E.altaica* (Trin.) Roshev. in Canada (see references in Taxonomy section). The geographic region with the most diversity of *Eremopoa* taxa is clearly Asia Minor; nearly all of the accepted species occur in Turkey.

There have been many differences of opinion on the species and infraspecific ranks to accept in *Eremopoa* (Table 1). Roshevitz (1934) treated six species in his new genus in the former U.S.S.R (*E.altaica*, *E.bellula* (Regel) Roshev., *E.oxyglumis* (Boiss.) Roshev., *E.multiradiata* (Trautv.) Roshev., *E.persica* and *E.songarica*). Tzvelev (1976) reduced these six species to two species, *E.persica* and *E.altaica*, with two and three subspecies, respectively, all of which were accepted as species by Czerepanov (1981, 1995). Scholz (1980, 1981) described two new species, *E.attalica* H. Scholz from Turkey and *E.medica* H. Scholz from Azerbaijan. The type of *E.medica* (holotype at W, isotype at B) was determined to be a species of *Puccinellia* Parl. (Soreng pers. obs. 2015). Mill (1985) treated six species in Turkey, including two new species, *E.capillaris* R.R. Mill and *E.mardinensis* R.R. Mill. Rahmanian et al. (2014) accepted four species in Iran, including *E.medica* and *E.persica* with three varieties.

**Table 1.** Classification history of *Eremopoa* and other taxa here accepted in Poasubg.Pseudopoa. Species and infraspecific taxa accepted by Roshevitz (1934) and authors of major floras and the region covered by their treatments are given. The last column provides the corresponding names in *Poa* accepted here.

Roshevits (1934)	Roshevits (in Köie 1945)	Bor (1970)	Tzvelev (1976, 1983)	Cope (1982)	Mill (1985)	Czerepanov (1995)	Zhu et al. (2006)	Gabrieljan and Oganesian (2010)	Rhamanian et al. (2014)	Euro+Med (on-line)	Here
**USSR**	**SW Iran**	**Iran, Afghanistan, w. Pakistan, n.w. Iraq, s. Turkmenistan, s.e. Azerbaijan**	**USSR**	**Pakistan**	**Turkey**	**USSR**	**China (Xinjiang, Xizang)**	**Armenia**	**Iran**	**Europe, Transcaucasia, Turkey, Levant, North Africa**	**whole range**
* E. persica *	* E. persica *	* E. persica *	* E. persica *	* E. persica *	* E. persica *	* E. persica *	–	* E. persica *	* E. persica *	* E. persica *	*** Poa persica ***
–	–	var. persica	subsp. persica	subsp. persica	–	–	–	–	var. persica	–	**subsp. persica**
–	var. major	–	–	–	–	–	–	–	–	–	–
* E. multiradiata *	–	(= var. songarica)	subsp. multiradiata	subsp. multiradiata	* E. multiradiata *	* E. multiradiata *	–	* E. multiradiata *	(= *persicavar.persica*)	* E. multiradiata *	***subsp. multiradiata***
* E. altaica *	–	–	* E. altaica *	* E. altaica *	–	* E. altaica *	* P. diaphora *	–	–	* E. altaica *	*** P. diaphora ***
–		–	subsp. altaica	subsp. altaica	–	–	subsp. diaphora	–	–	subsp. altaica	***subsp. diaphora***
											***var. diaphora***
* E. songarica *	–	var. songarica	subsp. songarica	subsp. songarica	* E. songarica *	* E. songarica *	–	* E. songarica *	var. songarica	subsp. songarica	***var. songarica***
* E. bellula *	–	* E. bellula *	(pp. = *altaica*, pp = *songarica*)	(= *altaica* s.l.)	(indirectly referenced, not accepted)	(pp. = *altaica*, pp = *songarica*)	–	–	* E. bellula *	–	(= var. alpina?)
P.persicavar.alpina (under *oxyglumis*)		–	–	–	(indirectly referenced, not accepted)	–	–	–	–	–	***var. alpina***
* E. oxyglumis *	* E. oxyglumis *	(= var. songarica)	subsp. oxyglumis	subsp. oxyglumis	(=*E.songarica*)	* E. oxyglumis *	subsp. oxyglumis	* E. oxyglumis *	E. persica var. oxyglumis	subsp. oxyglumis	**subsp. oxyglumis**
–	–	–	–	–	* E. attalica *	–	–	–	–	* E. attalica *	*** P. attalica ***
–	–	–	–	–	* E. capillaris *	–	–	–	–	* E. capillaris *	*** P. millii ***
–	–	–	–	–	* E. mardinensis *	–	–	–	–	* E. mardinensis *	(= P.persicasubsp.multiradiata)
–	* E. nephelochloides *	–	–	–	*E.nephelochloides* (Iran)	–	–	–	* E. nephelochloides *	–	*** P. nephelochloides ***
–	–	–	–	–	–	–	–	–	* E. medica *	–	(= *Puccinellia* sp.)
–	–	–	–	–	–	–	–	–	–	–	*** P. sintenisii ***
–	–	–	–	–	–	–	–	–	–	–	*** P. speluncarum ***

Bor’s genus *Lindbergella* (Bor 1968b, 1969) comprises a single annual species that is morphologically similar to *Eremopoa*. It differs from *Eremopoa* only in having firmer lemmas that are 3-veined and obscurely apiculate and panicles with 1–5 branches that are smooth. *Lindbergellasintenisii* (H. Lindb.) Bor was originally published as *Poasintenisii* by Lindberg (1942) and also as P.persicavar.cypria by Samuelsson (1950), the type of which is a syntype of P.persicavar.alpinaBoissier (1884). The species is endemic to Cyprus.

The first molecular data on *Eremopoa*, generated by our lab in 2004/2005, indicated that *E.songarica* was nested within *Poa*. That data was first published by Gillespie et al. (2007) using chloroplast DNA sequences from the *trnT-trnL-trnF* region. Based on this same data, inclusion of *Eremopoa* in *Poa* was already applied in the Flora of China account (Zhu et al. 2006, as P.subg.Pseudopoa (K. Koch) Stapf) and was continued in Gillespie et al. (2008, 2010), Soreng (2004+) and Soreng et al. (2010, 2015a, 2017a). Although nested within *Poa*, *Eremopoa* was positioned on a long branch separate from other *Poa* clades, justifying its recognition as a distinct subgenus, P.subg.Pseudopoa (Gillespie et al. 2007).

We published our initial DNA results for only one species of *Eremopoa* (*E.songarica*) based on *trnT-trnL-trnF* and, subsequently, nuclear ribosomal (nrDNA) ITS and ETS sequence data (Gillespie et al. 2007, 2008, 2010, Soreng et al. 2010). We subsequently sequenced two additional plastid regions (*matK* and *rpoB-trnC*) and added data for *Eremopoapersica* (Cabi et al. 2017, as *Poapersica*). A DNA analysis of ITS sequence data by Hoffmann et al. (2013) showed *Lindbergellasintenisii* was also nested within *Poa* near *Eremopoa*. Since then, we have accumulated nrDNA and plastid sequence data for most of the *Eremopoa* taxa and *L.sintenisii* and sampled many more species of *Poa* from Turkey and around the world. Analysis of our accumulated phylogenetic data on *Eremopoa* is presented here. All *Eremopoa* taxa were nested well within *Poa*, and *P.speluncarum* J.R. Edm. and *L.sintenisii* were found to be nested within or sister to the set of *Eremopoa* species. Here we place these taxa in Poasubg.Pseudopoa and present a taxonomic synopsis of all the species and infraspecies, as well as a key to the taxa we currently accept. Further study is needed before a comprehensive revision of the subgenus can be produced.

## Methods

Collections of *Eremopoa* at E and G (except those not available for loan), several from P and two type specimens from BM and B were loaned to RJS at US. Other material was examined by RJS at B, K, LE, P, US and herbaria in Turkey (ANK, ISTE, NKU). Fieldwork in which 38 specimens of *Eremopoa* were collected by us was conducted in Kyrgyz Republic (RJS 2006) and Turkey (RJS and associates 1994, 2013, 2014, 2015; LJG & RJS and associates 2011; EC was a co-collector on the 2011 to 2015 expeditions). Additional material was obtained from R. Hand (*Lindbergellasintenisii*) and M. Assadi and M. Amini-Rad (Iranian *Eremopoa*).

The molecular phylogenetic analysis included 77 samples: 15 *Eremopoa*, 56 *Poa*, 1 *Lindbergella* and 5 outgroup samples (Appendix 1). A diverse set of *Poa* species was chosen to represent the majority of sections, including all sections in southwest Asia. Outgroup taxa were chosen to include representatives of the two taxa (*Phleum* L. and *Milium* L.) and one clade considered most closely related to *Poa* (Gillespie et al. 2010, Soreng et al. 2015b). Sequences of *Lindbergella* and the majority of *Eremopoa* samples, plus many *matK* and *rpoB* sequences, are new to this study (Appendix 1). For simplicity, due to the confusing taxonomy and nomenclature, we refer to *Eremopoa* taxa using names at the species level in the Results, trees and Appendix 1 (see Table 1 for their corresponding names in *Poa*). The collection *TARI 135082* was previously identified as *E.medica* (Rahmanian et al. 2014), but was re-determined by RJS as P.persicasubsp.persica.

DNA was extracted from silica gel dried or herbarium leaf material as described in Gillespie et al. (2008). Three plastid markers (*matK, rpoB-trnC* and *trnT-trnL-trnF* [TLF]) and two nuclear ribosomal DNA (nrDNA) markers (internal transcribed spacer [ITS] and external transcribed spacer [ETS]) were sequenced. Amplification and sequencing protocols, including primers used, were described in our previous studies, as follows: ITS and TLF (Gillespie et al. 2008); ETS (Gillespie et al. 2009, 2010); *matK* and *rpoB-trnC* (Soreng et al. 2015b). Sequences were assembled, edited, aligned and concatenated using Geneious ver. 6.1.5 (http://www.geneious.com). The MAFFT ver. 7.017 plugin (Katoh and Standley 2013) was used for alignment, followed by manual adjustment. All samples are complete for all markers, except for several samples with missing ends. The molecular study was conducted at the Canadian Museum of Nature; sequencing was mostly performed by NA, analyses by LJG.

Maximum parsimony (MP) analyses were performed in PAUP* 4.0b10 (Swofford 2002) using the heuristic search command with default settings, including tree bisection-reconnection (TBR) swapping, saving all multiple shortest trees (Multrees) with a maximum number set to 100,000. Branch support was assessed using MP bootstrap analyses performed in PAUP* with heuristic search strategy, 10,000 bootstrap replicates, each with ten random addition sequence replicates, saving ten trees per replicate.

Bayesian Markov chain Monte Carlo analyses were conducted in MrBayes (Ronquist et al. 2011). Optimal models of molecular evolution for individual markers were first determined using the Akaike information criterion (AIC; Akaike 1974) conducted through likelihood searches in jModeltest with default settings (Darriba et al. 2012). Models were set at GTR + Γ for ITS, ETS and *rpoB-trnC* partitions and GTR + I + Γ for *matK* and TLF partitions based on the AIC scores and the models allowed in MrBayes. Two independent runs of four chained searches were performed for either two or three million generations (analyses were stopped when split frequency of 0.005 was reached or closely approached), sampling every 500 generations, with default parameters. A 25% burn-in was implemented prior to summarising a 50% majority rule consensus tree and calculating Bayesian posterior probabilities (pp).

MP heuristic searches and bootstrap analyses were performed initially on the separate marker alignments. Strict consensus trees were examined for conflicting topologies with incongruence identified by branch conflicts with ≥75% bootstrap support (BS). No supported incongruence was found between ITS and ETS trees, nor amongst the three plastid trees. Further MP and Bayesian analyses were performed on the separate concatenated nrDNA (77 samples, 1251 aligned characters) and plastid (77 samples, 4465 characters) alignments. Since supported incongruence was detected between the nrDNA and plastid strict consensus trees, species and clades determined to be incongruent were removed prior to performing analyses on the concatenated combined nrDNA and plastid alignment (68 samples, 5599 aligned characters). Trees were viewed in FigTree v1.4.0 (Rambaut 2006+). Clade designations follow Soreng et al. (2010) with modifications as in Cabi et al. (2017) and Soreng et al. (2017b), wherein well-supported major clades are assigned letters.

## Results

**Figure 1.**
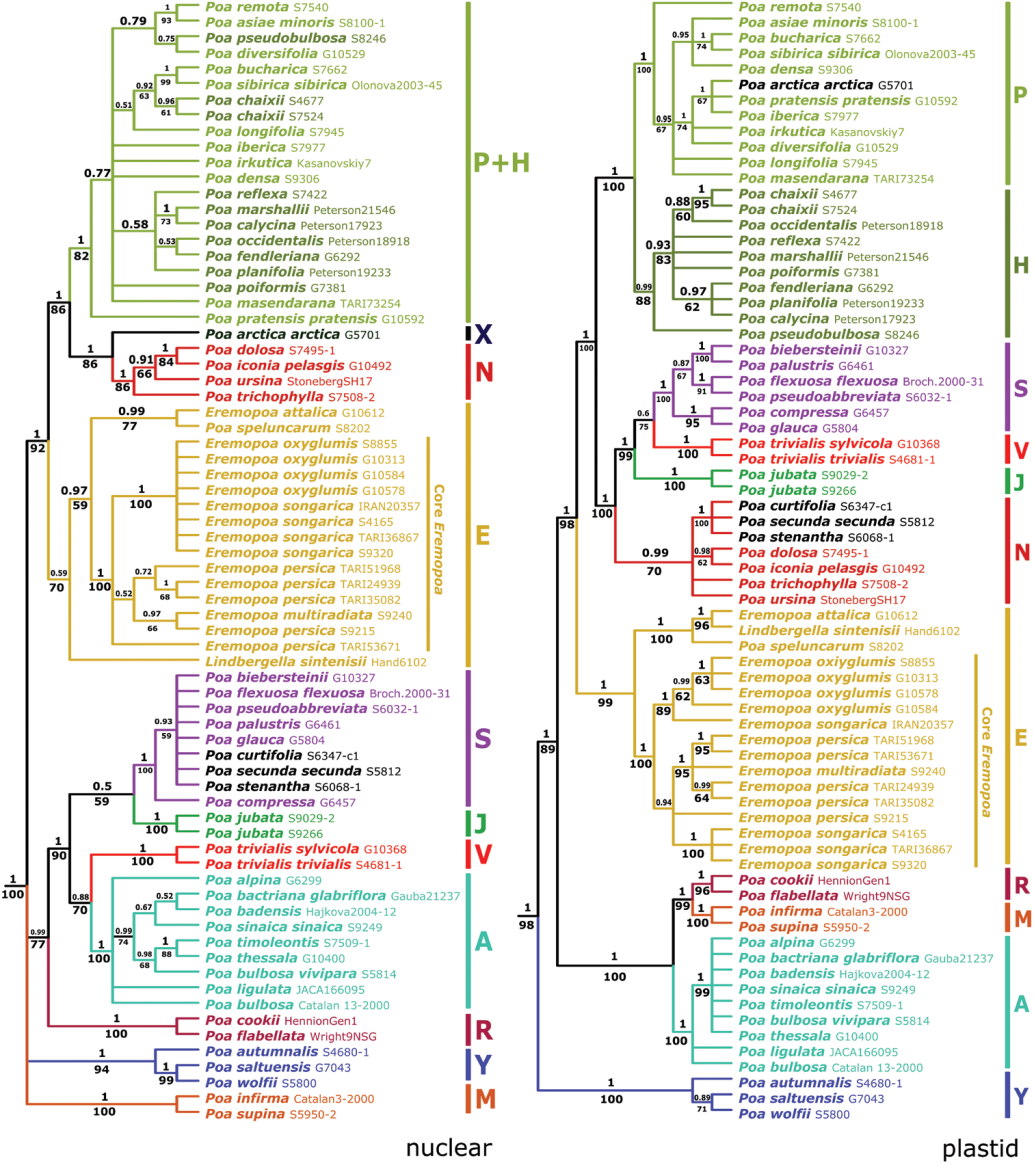
*Poa*nrDNA and plastid Baysian analyses showing placement of *Eremopoa* and *Lindbergella*. Bayesian 50% majority rule consensus trees of nrDNAITS and ETS (left) and plastid data (*trnT*-*trnL*-*trnF*, *matK* and *rpoB*-*trnC*) (right). Bayesian posterior probabilities are shown above branches, MP bootstrap values below branches. Outgroups are not shown. Major clades are indicated by colour and capital letter. Taxa shown in black belong to different major clades in plastid and nrDNA trees.

Plastid and nrDNA Bayesian trees are given in Fig. 1 with summary statistics in Suppl. material 1. There are 100 new sequences reported in GenBank and these are given in Appendix 1. MP trees (bootstrap values shown below branches in Fig. 1) were very similar to the Bayesian trees with a few minor unsupported differences. Major clades (shown by letter and colour in Fig. 1) are identical in both nrDNA and plastid trees, with two exceptions: *Poaarctica* R. Br. and P.sect.Secundae members (*P.curtifolia* Scribn., *P.secunda* J. Presl and *P.stenantha* Trin.), each belonging to different major clades in the two trees. The position of three major clades differs significantly between the nrDNA and plastid trees: **J** clade (sect. Jubatae: *P.jubata* A. Kern.), **S** clade (sects. *Stenopoa* and *Abbreviatae*) and **V** clade (sect. Pandemos: *P.trivialis* L.). *Poa* major clades have been described elsewhere (Gillespie et al. 2007, 2008, 2009, Soreng et al. 2010, 2017b, Cabi et al. 2017); here we focus on the position of *Eremopoa*.

*Eremopoa* species, together with *Lindbergellasintenisii* and *Poaspeluncarum*, form a clade (**E** clade) in both nrDNA and plastid trees, but are strongly supported only in the plastid analysis (pp = 1, BS = 99%). All *E.multiradiata*, *E.oxyglumis*, *E.persica* and *E.songarica* accessions form a strongly supported clade (core *Eremopoa* clade) in both trees (pp = 1, BS = 100%). In the plastid analysis *E.attalica*, *L.sintenisii* and *P.speluncarum* form a strongly supported clade (pp = 1, BS = 100%), with *L.sintenisii* sister to *E.attalica* (pp = 1, BS = 96%). In the nrDNA tree, *E.attalica* and *P.speluncarum* are sister taxa (pp = 0.99, BS = 77%) and *Lindbergella* is weakly supported as sister to this clade plus the core *Eremopoa* clade (pp = 0.97, BS = 59%). Within the core *Eremopoa* clade, all *E.oxyglumis* and *E.songarica* samples form a strongly supported clade in the nrDNA analysis (pp = 1, BS = 100%), whereas in the plastid analysis, these samples are divided between two strongly supported clades corresponding to *E.oxyglumis* plus one *E.songarica* sample (*IRAN 20357*, identification needs confirmation) (pp = 1, BS = 89%) and all remaining samples of *E.songarica* (pp = 1, BS = 100%). *Eremopoamultiradiata* and *E.persica* samples do not form a clade in either analysis, although all except one (*E.persica*, *Soreng 9215*) are strongly supported as a clade (pp = 1, BS = 95%) in the plastid tree.

**Figure 2.**
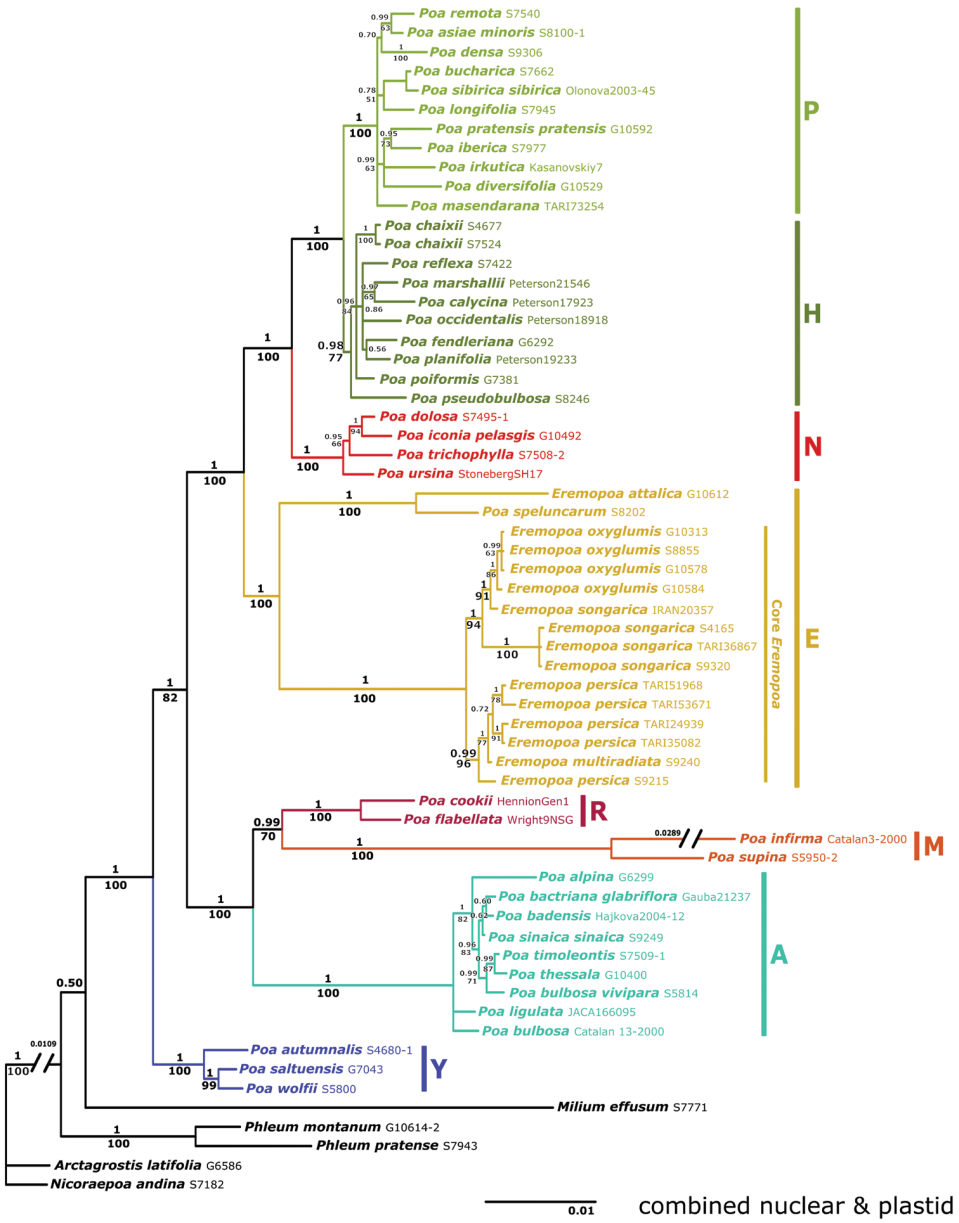
*Poa* combined nrDNA and plastid Baysian analysis showing placement of *Eremopoa*. Bayesian 50% majority rule consensus tree of combined nrDNA (ITS and ETS) and plastid data (*trnT-trnL-trnF, matK and rpoB-trnC*). Bayesian posterior probabilities are shown above branches, MP bootstrap values below branches. Major clades are indicated by colour and capital letter; outgroups are shown in black.

The combined nrDNA and plastid Bayesian tree with proportional branch lengths is shown in Fig. 2. Prior to analysis, species and clades with positions incongruent (branch conflicts with ≥ 75% BS) between the nrDNA and plastid trees were removed, including *Lindbergellasintenisii*, *P.arctica*, P.sect.Secundae species and the **J**, **S**, and **V** clades. The **E** clade is strongly supported, as are its two subclades, *E.attalica*-*P.speluncarum* and the core *Eremopoa* clade (all pp = 1, BS = 100%). Both subclades are on long branches and separated by considerable genetic distance. The core *Eremopoa* clade is subdivided into two strongly supported clades: *E.multiradiata*-*E.persica* (pp = 0.99, BS = 96%) and *E.oxyglumis*-*E.songarica* (pp = 1, BS = 94%). *Eremopoaoxyglumis* and three of four accessions of *E.songarica* each form moderately or strongly supported clades (pp = 1, BS = 86%; pp = 1, BS = 100%, respectively).

In the combined nrDNA and plastid tree (Fig. 2), the **E** clade is strongly supported as sister (pp = 1, BS = 100%) to a clade comprising *Poa* supersects. *Homalopoa* (**H** clade) and *Poa* (**P** clade) and the **N** clade (P.sect.Nanopoa plus unassigned species). In the nrDNA analysis, the **E** clade is strongly supported as sister to clades **P+H** (not differentiated), **N**, and **X** (represented here by *P.arctica*) (Fig. 1). In the plastid analysis, the **E** clade is sister to a larger clade comprising clades **H**, **N**, and **P**, plus **J**, **S** and **V** (Fig. 1).

## Discussion

Our molecular analyses of plastid and nuclear ribosomal DNA strongly support the position of *Eremopoa* and *Lindbergella* within the genus *Poa.Eremopoa* and *Lindbergella* were united in a clade along with *Poaspeluncarum* with strong support in the plastid and combined trees (weak support in the nuclear tree). We call this set the **E** clade (Soreng et al. 2010, Cabi et al. 2017) and accept it as Poasubg.Pseudopoa. In its recent usage, this subgenus was initially considered to include only *Eremopoa* (Zhu et al. 2006, Gillespie et al. 2007); here it is expanded to include *Lindbergella* and *P.speluncarum*.

Within the **E** clade, three taxa of southwest Turkey and Cyprus, *E.attalica*, *P.speluncarum* and *Lindbergellasintenisii*, are phylogenetically isolated from all the other species of *Eremopoa* sampled (the core *Eremopoa* clade). All three taxa formed a strongly supported clade in the plastid tree, while in the nuclear tree only the first two species form a clade and *L.sintenisii* is sister to this clade plus the core *Eremopoa* clade. The position of *L.sintenisii* is moderately supported as incongruent between the nuclear and plastid trees suggesting that the genus may be of hybrid origin; however, further studies are needed to confirm incongruence over lack of support.

All *Eremopoa* taxa sampled, excluding *E.attalica*, form a strongly supported clade in all trees, called here the core *Eremopoa* clade. This clade includes two strongly supported subclades in the combined nuclear-plastid tree, corresponding to *E.persica* s.l. and *E.altaica* s.l. In the first subclade, *E.multiradiata* is nested amongst *E.persica* samples, as is the sample originally determined as *E.medica* (*TARI 35082*). The *E.multiradiata* sample (*Soreng 9240*) comes from the type locality of *E.mardinensis* in SW Turkey and is a good match for that species, but we believe that *E.mardinensis* should be treated as a synonym of *E.multiradiata*. The *E.altaica* s.l. subclade in the combined tree includes a strongly supported and divergent clade of three *E.songarica* samples and a clade of *E.oxyglumis* plus one sample of *E.songarica* (identification needs confirmation). The position of *E.songarica* (tetraploid) with *E.oxyglumis* (diploid and hexaploid) is strongly supported in the combined and nuclear trees, but is weakly supported with *E.persica* (diploid) in the plastid tree. This, together with ploidy level, is suggestive of a possible hybrid origin for *E.songarica*, but this hypothesis needs to be further explored.

As noted in the introduction and Table 1, there has been no consensus on the taxonomy of *Eremopoa* species. Bor (1970, p. 49) wrote “As far as the genus *Eremopoa* Roshev. is concerned I am prepared to accept two species only: *Eremopoapersica* (Trin.) Roshev. and *E.bellula* (Regel) Roshev.” He considered *E.songarica*, *multiradiata* and *oxyglumis* “only worthy of varietal rank” as the single taxon, E.persicavar.songarica. Tzvelev (1976), Cope (1982) and Mill (1985) dismissed the *E.bellula* form as indistinct, yet it was maintained as a species by Bor (1970) and Rahmanian et al. (2014). As such, the array of taxa has been treated as a series of species, subspecies or varieties. The taxonomy proposed by Tzvelev (1976) seems the most useful for treating *E.persica* s.l. and *E.altaica* s.l.; each is treated as a separate species with subspecies. His classification, supported by molecular data, is adopted here with some minor modifications.

Here, we present a synopsis of P.subg.Pseudopoa based on our current understanding. Further herbarium and molecular study is needed before a more comprehensive revision of the subgenus can be produced. We treat all *Eremopoa* species, *Lindbergellasintenisii* and *P.speluncarum* in P.subg.Pseudopoa. We merge all *Eremopoa* taxa and *L.sintenisii* into *Poa* and treat the *Eremopoa* taxa as five species. *Poadiaphora* Trin. is the correct name for *E.altaica* within Poa. Two subspecies, subsp. diaphora and *oxyglumis* (Boiss.) Soreng & G.H. Zhu, are recognised in *P.diaphora* based in part on their mostly clear separation in the plastid analyses and morphological distinctions. Subspecies diaphora includes three difficult to distinguish varieties: var. diaphora (formerly *E.altaica*), var. alpina and var. songarica (formerly *E.songarica*). *Poapersica* includes two subspecies and is clearly separated from both *P.diaphora* subspecies in the analyses. Most *Eremopoa* taxa already have names in *Poa* or the epithets used in *Eremopoa* are available in *Poa* (with one exception).

## Taxonomy

### Poa subg. Pseudopoa

Taxon classificationPlantaePoalesPoaceae

(K. Koch) Stapf in J. D. Hooker, Fl. Brit. India 7(22): 337. 1897 [1896].

Festuca [unranked] Pseudopoa K. Koch, Linnaea 21(1[4]): 409. 1848. Poasect.Pseudopoa (K. Koch) Hack., Nat. Pflanzenfam. 2(2): 73. 1887. Eremopoa Roshev., Fl. URSS 2: 429, 756. 1934. Type. Poapersica Trin. ≡ Festucapersica (Trin.) K. Koch.
Lindbergia Bor, Svensk Bot. Tidskr. 62: 467, 1968 (nom. illeg. hom., non Kindb., 1897). Lindbergella Bor, Svensk Bot. Tidskr. 63: 368. 1969. Type. Poasintenisii H. Lindb. ≡ Lindbergellasintenisii (H. Lindb.) Bor.

#### Emended diagnosis.

Like species of other *Poa* subgenera, but annual (*P.speluncarum* a weak stooling perennial) and differing from other annual species of *Poa* by combination of sheath margins fused only near the base (basal sheaths fused to 16%, top sheath 4–12% [to 50% in *P.speluncarum*]), panicle branches scabrous along angles (*P.sintenisii* smooth), arranged in whorl-like groups of 5 to 27 per node (sometimes fewer in *P.diaphora* and *P.sintenisii*), sometimes the lower whorls of branches naked or with only a few sterile spikelets, flowers bisexual, glumes short (lower glume 2/7–2/3 (–3/4) the first lemma in length), 1-veined (3-veined in *P.sintenisii*), apex sharply pointed, sometimes apiculate, rachilla internodes exposed, scaberulous, callus glabrous (or with a short crown of hairs in *P.sintenisii*), lemmas membranous to subchartaceous (*P.sintenisii* chartaceous), 3–5 veined, the intermediate veins faint when present, laterally compressed, but the keel not pronounced, glabrous or keel and marginal veins short sericeous (also sericeous between the veins in *P.sintenisii*), but keel scabrous distal to the hairs.

#### Distribution.

Southwest Asia from Israel, Lebanon, Cyprus and Turkey eastwards through Transcaucasia, Iran, central Asia to western China and northwest India. Sporadic elsewhere, possibly adventive on Egypt’s North African coast but native east of the Red Sea, adventive in Europe and Canada.

#### Notes.

A subgenus of seven species with several infraspecies, distributed mainly in semi-arid midlands to uplands (usually 300 m plus) to alpine, with winter spring / summer drought precipitation pattern, often along trails and roads, cultivated fields and pastures, around puddles, shallow springs, swales and vernal pools, snow beds, in pine/oak forests to open grasslands and deserts, also in shallow caves, in shallow sandy or stony soils or screes of igneous or metamorphic rocks of igneous or sedimentary origin, including pumice, lava, serpentine, shale, sandstone, limestone and marble.

##### Key to Poasubgen.Pseudopoa taxa and other annual species of *Poa* in the coincident geographic region

Plants annual (infrequently perennial or perenniating); anthers mostly 0.2–1 mm (to 1.7 mm in the weak stemmed, stooling perennial ***P.speluncarum***, to 2.8 mm in the annual species ***Poapersica***).

**Table:** 

1	Palea keels soft hairy, never scabrous; callus glabrous (Poasect.Micrantherae)	**2**
–	Palea keels scabrous at least in part (if hairy in part, then distally scabrous); callus glabrous or hairy	**3**
2	Anthers 0.2–0.5 mm long; panicle branches ascending, spikelets congested along the branches; plants light green	***Poainfirma* Kunth**
–	Anthers 0.5–1 mm long; panicle branches spreading to ascending, spikelets moderately congested along the branches; plants darker green	***Poaannua* L.**
3	Spikelets ovate; lemma keels densely villous medially, many hairs over 0.5 mm long; callus with a plicate web; anthers 0.4–0.8 mm long; panicles short (to 5 cm long), branches terete, smooth or sparsely scabrid, with 1–2 branches per node; upper culm sheath margins fused 25–35(–50)% their length; plants of vernal swales, Albania, Croatia, Greece, Bulgaria and European part of Turkey **(Poasect.Jubatae)**	*** Poa jubata ***
–	Spikelets generally lanceolate; lemma keels glabrous or sericeous, hairs less than 0.3(–0.5) mm long; callus glabrous or with a short crown of hairs; anthers 0.2–2.8 mm long; panicles short or long, branches angled, smooth or scabrous, mostly with 2 to 27 branches per node, commonly appearing whorled; upper culm sheath margins fused 4–12% their length (40–50% in *P.speluncarum*); plants of Cyprus, Anatolian Turkey, southwards and eastwards across Asia into China (**Poasubg.Pseudopoa, incl. *Eremopoa*)**	**4**
4	Uppermost culm sheath margins fused 40–50% their length; spikelets mostly 1-flowered; lemmas glabrous; callus glabrous; anthers 1.1–1.7 long; plants feeble, stooling perennials of caves and shady cool moist places in the Taurus Mts. of Turkey (rare) (**Poasect.Speluncarae**)	*** Poa speluncarum ***
–	Uppermost culm sheath margins fused 4–12% their length; spikelets (1–)2 to 10-flowered; lemmas glabrous or pubescent; callus glabrous or with a minute crown of hairs; anthers 0.2–2.8 mm long; plants slender tufted annuals	**5**
5	Lemmas 3-veined, apex slightly apiculate, lemmas and paleas subcoriaceous, sericeous along the keel(s) and marginal veins and between the veins; panicle branches smooth, mostly 1–5 at lower nodes; callus glabrous or with a short crown of hairs; plants endemic to Cyprus (usually on serpentine substrates) (**Poasect.Lindbergella**)	*** Poa sintenisii ***
–	Lemmas 5-veined (veins commonly faint), apex infrequently apiculate, lemmas and paleas subchartaceous to subcoriaceous, glabrous between the veins or throughout; panicle branches scabrous, (1–)5–27 at lower nodes; callus glabrous; plants widespread, but not in Cyprus (**Poasect.Pseudopoa)**	**6**
6	Panicles with 1 to 3 lower whorls of 7 or more sterile/naked or mostly sterile branches; panicles 7–20 cm long, effusely branched; lemmas 2–2.5 mm long, sericeous along the keel and marginal veins; spikelets 1–4(–6)-flowered	**7**
–	Panicles not or infrequently with some sterile lower branches; panicles 2–21 cm long, effusely to sparsely branched; lemmas 1.8–4.5 mm long, glabrous or sericeous along the keel and marginal veins; spikelets 1–12-flowered	**8**
7	Anthers 1.1–1.5 mm long; ligules 1.5–2.5 mm long; branches 7–20 per lower whorl; spikelets 1–4(–6)-flowered; plants of Zagros Mts., Iran	*** P. nephelochloides ***
–	Anthers 0.8–1 mm long; ligules 1–1.5 mm long; branches 7–15 per lower whorl; spikelets 1–3-flowered; plants of Taurus Mts., Turkey	*** P. attalica ***
8	Anthers (1.2–)1.4–2.8 mm long; lemma apex blunt or obtuse to acutely pointed, with a broad membranous margin **(*P.persica* s.l.)**	**9**
–	Anthers 0.2–1.3 mm long; lemma apex acute or narrowly acute to acuminately pointed, with a narrow membranous margin (blunt or slightly pointed in *P.millii* but then with 13–27 branches at lower panicle nodes)	**10**
9	Lemmas all glabrous or rarely with a few hairs near the base of the keel or marginal veins; spikelets (4–)5–10(–12)-flowered; panicles usually ¼–½ the plant height; anthers 1.5–2.8 mm long	** P. persica subsp. multiradiata **
–	Lemmas (at least of the lowest flower in a spikelet) minutely sericeous along the keel and marginal veins for ¼–⅔ the length; spikelets (2–)3–7(–9)-flowered; panicles usually ⅖–⅔ the plant height; anthers (1.2–)1.4–1.8 mm long	** P. persica subsp. persica **
10	Anthers mostly 0.2–0.6 mm long; lemmas 1.8–4.5 mm long, apex sharply pointed, usually glabrous, infrequently sparsely puberulent along the keel with one or a few soft hairs scattered near the base; spikelets (1–)2–3(–5)-flowered; plants 2–40 cm tall	**11**
–	Anthers 0.6–1.3 mm long; lemmas 2.3–3 mm long, apex acute and sharply pointed to obtuse and blunt, at least the lowest lemma in a spikelet evenly sericeous (hairs ca. 0.1–0.3(–3.5) mm long, stiff, appressed) along the keel in the proximal ¼–½ and along the marginal veins near the base; spikelets 3–5(–9)-flowered; plants mostly 15–60 cm tall	**13**
11	Lemmas 3.5–4.5 mm long; panicles (2–)3–8(–9) cm long, branches 1–5(–7) at lower nodes, divaricately rebranched and relatively stout, spikelets usually sparse and few; plants mostly 5–25(–30) cm tall	** P. diaphora subsp. diaphora var. diaphora **
–	Lemmas 1.8–3.5(–3.8 in large specimens with many spikelets) mm long; panicles 2–15(–20) cm long, branches (1–)3–8(–12) at lower nodes, divaricately rebranched or not, capillary to somewhat stout, spikelets sparse to crowded, few to many; plants 2–40 cm tall	**12**
12	Plants low growing, with dense fascicles of rebranching culms; culms 2–6 cm tall, with lateral inflorescences from lower culm leaves; panicles contracted to open, 1.5–4 cm long, included in tuft of basal leaves or slightly exerted; lemmas 3–3.5 mm long; plants alpine	** P. diaphora subsp. diaphora var. alpina **
–	Plants low growing or taller, without fascicles of rebranching culms; culms solitary to several, mostly 10–40 cm tall, without lateral inflorescences; panicles effuse, usually more than 5 cm long, usually exerted; lemmas 1.8–3.5 mm long; plants of various habitats	** P. diaphora subsp. diaphora var. songarica **
13	Spikelet pedicels mostly 2–5 mm long; panicle branches 5–18 at lower nodes, stiffly spreading, lower whorls never naked or with rudimentary spikelets; lemma apices acutely pointed; anthers 0.6–1.0(1.1) mm long	** P. diaphora subsp. oxyglumis **
–	Spikelet pedicels mostly 5–10 mm long: panicle branches (9–)13–27 at lower nodes, slender, slightly flexuous, lower whorls sometimes with a few branches that are naked or with some rudimentary spikelets in addition to normal spikelets; lemma apices obtuse to acute, blunt or slightly pointed; anthers 0.8–1.3 mm long	*** P. millii ***

### Poa subg. Pseudopoa sect. Pseudopoa

Taxon classificationPlantaePoalesPoaceae

(K. Koch) Hack., Nat. Pflanzenfam. 2(2): 73. 1887.

#### Emended description.

Tufted annuals. Leaf sheaths keeled, margins fused for 4–12% their length; blades flat to convolute, surfaces scabrous. Panicles open, with (1–)3–27 branches at lower nodes, lower whorls sometimes sterile; branches ascending to widely spreading, scabrous angled, with pedicels mostly equalling or up to 4× longer than their spikelets. Spikelets 1–10-flowered; glumes unequal, 1^st^ glume 1-veined, 2^nd^ glume 3-veined, usually reaching to less than ⅔ the adjacent lemma; rachilla internodes terete, scabrous; callus smooth, glabrous, with a round disarticulation scar; lemmas laterally compressed, weakly keeled, glabrous or short sericeous in lower half of the keel and also along the marginal veins, between veins smooth or scabrous, glabrous (rarely sericeous), 5-veined, intermediate veins obscure to distinct, margins narrowly to broadly scarious, apex obtuse to acuminate, sometimes briefly muticus. Flowers perfect, ovaries glabrous, anthers 0.2–2.8 mm long; caryopsis 1.5–2.5 mm long, narrowly elliptical, laterally compressed, fused to the palea, solid, hilum ⅛–⅙ the grain in length.

### Poa attalica

Taxon classificationPlantaePoalesPoaceae

(H. Scholz) Soreng, Cabi & L.J. Gillespie comb. nov.

urn:lsid:ipni.org:names:77191831-1

Eremopoa attalica H. Scholz, Willdenowia 10(1): 33, f. 1. 1980.

#### Type.

Turkey. Antalya, “nordwestl. Antalya bei Termessos, ausgetrockneter Gebirgsbach”, 300 m, 23 Jul 1979, *Kehl s.n.* (holotype: B! [B-100272775])

#### Distribution.

Turkey (western Taurus Mts.).

#### Notes.

We provisionally retain this species in sect. Pseudopoa, despite its divergent phylogenetic placement. The species is morphologically similar to other members of the section. As noted by Mill (1985), it is most like *Poanephelochloides* Roshev., but the anthers are smaller. Some populations of *P.millii* approach *P.attalica* and are problematical to separate (see under *P.millii*). Further molecular study is needed to determine if the three species are closely related and if a new section is warranted.

### Poa diaphora

Taxon classificationPlantaePoalesPoaceae

Trin., Mém. Acad. Imp. Sci. St.-Pétersbourg, Sér. 6, Sci. Math., Seconde Pt. Sci. Nat. 4,2(1): 69–70. 1836.

Aira altaica Trin., Mém. Acad. Imp. Sci. St.-Pétersbourg Divers Savans 2: 526. 1835. Nephelochloaaltaica (Trin.) Griseb., Fl. Ross. 4(13): 367. 1852. Poadiaphana Boiss., Fl. Orient. 5: 611. 1884, nom. inval. Eremopoaaltaica (Trin.) Roshev., Fl. URSS 2: 431. 1934.

#### Type.

“Sterilissimus salsuginosis deserti editi Tschujae”, [1800–3000 m], July 1832, *A. Bunge* (lectotype, designated by Tzvelev 1976, pg. 480, and marked in herbarium: LE! [Trinius herbarium microform image 424-A4! p.p. Bunge 1832]; isolectotypes: LE [3 specimens: TRIN-2620.01! with original description (Trinius herbarium microform 312-A1), Trinius herbarium microform images 424-A3!, 424-A5!], K [K000789849 image!; specimen labelled “Airaaltaica Trin. Altai”, “Acad. St. Petrop, mis. 8br 1835” is a good match for LE type material]). See Soreng et al. (1995) for explanation of Trinius herbarium citations.

#### Distribution.

Egypt (Sinai Peninsula) to China (Xinjiang, Xizang).

#### Notes.

Separating the four forms of *Poadiaphora* s.l. treated here is often difficult. Here we choose to recognise two subspecies as divided in the molecular plastid analysis. Subspecies diaphora and *oxyglumis* are most easily separated by the minute anthers (0.2–0.6 mm) combined with glabrous or nearly glabrous lemmas in the former and slightly longer anthers (0.6–1.1 mm) combined with hairy lemma keels and marginal veins in the latter. The other forms, *diaphora* s.s., *songarica* and *alpina* are essentially intergrading and are here treated as varieties in subsp. diaphora.

The specimen K000789848 (image!) (“Al. Bunge” ex hrbr. Alexandri Lehmann, Reliquiae botanicae, original det “*Poadiaphora* Tr.”) might be original material of *Airaaltaica*, but RJS doubts it as it is not a good match for LE types; it is a taller plant more like K00789847 (also Reliquiae Lehmannianae), which is *Bunge* material collected 20 May 1842, in Karakum desert.

### Poa diaphora subsp. diaphora var. diaphora

Taxon classificationPlantaePoalesPoaceae

Fig. 3A

Poa persica var. diaphora (Trin.) Asch. & Graebn., Syn. Mitteleur. Fl. 2: 437. 1900. Eremopoaaltaica(Trin.)Roshev.subsp.altaica.

#### Distribution.

China (Xinjiang, Xizang), Kazakhstan, Kyrgyz Republic, Pakistan, Russia (Altai Mts.), Tajikistan, Turkey.

#### Notes.

A single specimen recorded from Turkey (Kars Prov., *Litvinov 4790* US ex K) evidently belongs to this variety and was also cited by Mill (1985) under *E.songarica*.

**Figure 3.**
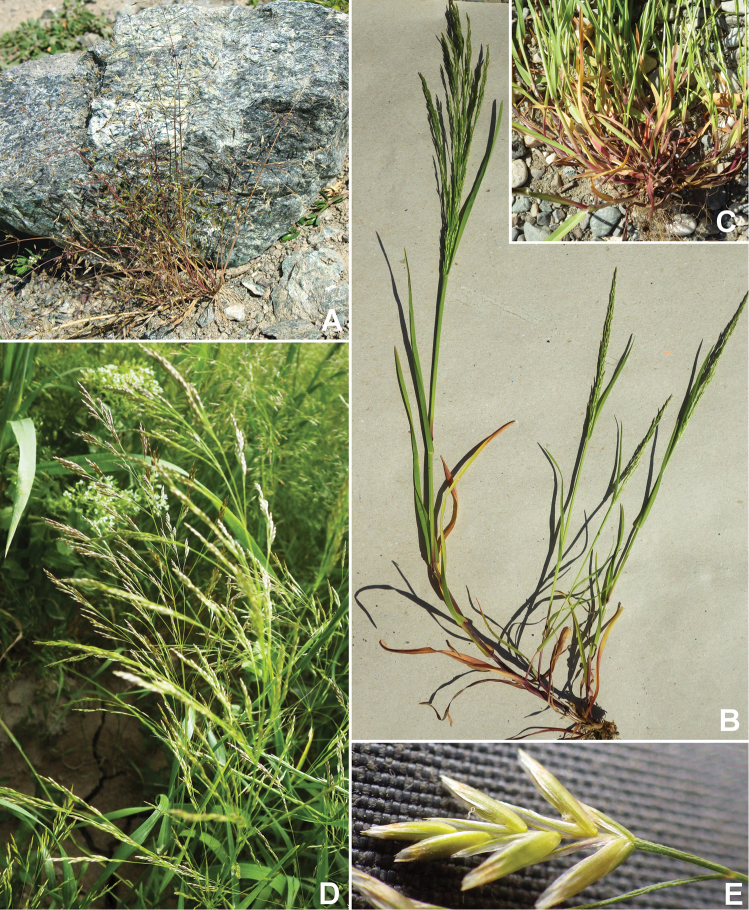
PoasubgenusPseudopoasect.Pseudopoa. **A**P.diaphorasubsp.diaphoravar.diaphora, Chu, Kyrgyz Republic (*Soreng et al. 7537*) **B, C**P.persicasubsp.persica, Adiyaman, Turkey (*Soreng et al. 9215*) **B** habit **C** closeup of base of plant showing keeled leaf sheaths and caniculate blades **D, E**P.persicasubsp.multiradiata, Mardin, Turkey (*Soreng et al. 9240*) **D** habit **E** spikelet showing glabrous lemmas. Photos by R.J. Soreng.

### Poa diaphora subsp. diaphora var. alpina

Taxon classificationPlantaePoalesPoaceae

(Boiss.) Soreng, Cabi & L.J. Gillespie comb. nov.

urn:lsid:ipni.org:names:77191833-1

Poa persica var. alpina Boiss., Fl. Orient. 5: 610. 1884.

#### Type.

Turkey. Plantae Lyciae, ad fonts reginis alpinae montis Elmalu, 25 Jun 1860, *E. Bourgeau 271* (lectotype, **here designated**: G [G00330280 image!]; isolectotypes: G [G00380172 image!, p.p. central and right top two samples], G [G0038173 image!], K [K-000789856 image!]).

#### Distribution.

Armenia, Azerbaijan, Afghanistan, Georgia, Iran, Kyrgyz Republic, Pakistan, Turkey and Turkmenistan(?).

#### Notes.

This taxon, accepted as *Eremopoabellula* by several authors (see Names of Uncertain Application below), was first recognised infraspecifically by Boissier (1884) as Poapersicavar.alpina. The variety is common in the highest elevations at which the genus occurs, in the alpine of Turkey, Iran and Afghanistan to the Pamir mountains, reaching 4000 m. Further study is needed to clarify the distinction of var. alpina from var. diaphora and these from *Eremopoabellula*, as the material placed here appears heterogenous.

Of the six syntypes of var. alpina cited by Boissier (*Bourgeau 271*, hab. in alpinis, montes supra Elmali Lyciae [G00380172, G0038173, G00330280, K000789856]; *Kotschy 12*, Tarus Cilicicus, 5–6000’; Prairies humides de la region alpine du Taurus, au Boulgarmden [as *12d*: G00330281, K000789851 image!]; *Balansa s.n.*, Jul-Aug 1855 [K000789857, P02358251 p.p. bottom right]; *Blanche s.n*., Libani cacuminal; *Kotschy 477*, mons Kuh Delu Persiae australis, 10 Jun 1842 [BM000959359 image!, E!, G00308632 image!, P02358251! p.p. “fo. pygmaea” bottom left]), we select *Bourgeau 271* as the lectotype as it is typical of the form. As noted by Samuelsson (1950), the *Sintenis* syntype (mons Troodos, Cypri) represents a separate form that is treated here as *Poasintenisii*. *Poapersica* var. “*minor*” Boiss. (cited by Mill, in Fl. Turkey 9: 492. 1985) is a nomen nudum since it is a herbarium name on *Bourgeau 271*, syntype of var. alpina Boiss.; this name is also inscribed on *Kotschy 12d* (p.p. G00308174), but the latter is original material, not a syntype, mentioned by Boissier (1884).

### Poa diaphora subsp. diaphora var. songarica

Taxon classificationPlantaePoalesPoaceae

(Schrenk ex Fisch. & C.A. Mey.) Soreng, Cabi & L.J. Gillespie comb. nov.

urn:lsid:ipni.org:names:77191834-1

Glyceria songarica Schrenk ex Fisch. & C.A. Mey., Enum. Pl. Nov. 1: 1–2. 1841. Nephelochloasongarica (Schrenk ex Fisch. & C.A. Mey.) Griseb., Fl. Ross. 4(13): 367. 1852. Nephelochloapersicavar.songarica (Schrenk ex Fisch. & C.A. Mey.) Regel, Trudy Imp. S.-Peterburgsk. Bot. Sada 7: 603. 1881. Poasongarica (Schrenk ex Fisch. & C.A. Mey.) Boiss., Fl. Orient. 5: 611. 1884. Poapersicavar.songarica(Schrenk ex Fisch. & C.A. Mey.) Stapf, Fl. Brit. India 7(22): 337. 1897 [1896]. Eremopoasongarica (Schrenk ex Fisch. & C.A. Mey.) Roshev., Fl. URSS 2: 431, pl. 32, f. 11. 1934. Eremopoapersicavar.songarica (Schrenk ex Fisch. & C.A. Mey.) Bor, Grass. Burma, Ceylon, India & Pakistan 532. 1960. Eremopoaaltaicasubsp.songarica (Schrenk ex Fisch. & C.A. Mey.) Tzvelev, Bot. Zhurn. (Moscow & Leningrad) 51(8): 1104. 1966. Poadiaphorasubsp.songarica (Schrenk ex Fisch. & C.A. Mey.) Soreng & G.H. Zhu, Fl. China vol. 22: 266. 2006. Poasongaricavar.argaea Hausskn. & Bornm. ex R.R. Mill, Fl. Turkey & E. Aegean Isl. 9: 491. 1985, nom. inval., as syn. of Eremopoasongarica.
Poa paradoxa Kar. & Kir., Bull. Soc. Imp. Naturalistes Moscou 864. 1841, nom. illeg. hom. Poasubtilis Kar. & Kir., Bull. Soc. Imp. Naturalistes Moscou 15(3): 532. 1842. nom. nov. (cited Poaparadaxa Kar. & Kir., 1941 [entry no.] 926). Type protologue. Hab. in herbosis ad rivulum Ai deserti Soongoro-Kirghisici, Jun, *Karelin & Kiriloff*. Type: Hab. in herbosis ad rivulum Ai deserti Soongoro-Kirghisici, Jun 1840, *Karelin & Kiriloff* (Herb. Fischer no. *504*) (lectotype, **here designated**: LE!; isotypes: P [P02663383!], W [W0028251 image!]).

#### Type.

Ad fl. Karatal versus montes Karatau, 13 June 1840, *H. Schrenk* s.n. (holotype: LE; isotype: LE).

#### Distribution.

Afghanistan, Armenia, Azerbaijan, China (Xizang), Georgia, Iran, Israel, Kazakhstan, Kyrgyz Republic, Tajikistan, Turkey, Turkmenistan and Uzbekistan.

#### Notes.

Poadiaphoravar.songarica was recently recorded (as Eremopoasongarica; determination verified here) from one locality in northernmost Israel (Danin and Fragman-Sapir 2016+). It was collected as a waif in Canada (Manitoba) in the 1950s (Stevenson 1965, as E.persica; Darbyshire 2007, as E.altaica: re-identified here), but is apparently not persistent (Darbyshire 2007, B.A. Ford, pers. comm, 2018).

Tzvelev (1976, pg. 480) cited “In herbidis Songaria ad rivulum Tschulak [Jun 1841], *Karelin & Kiriloff 2123*” (LE!) as type of P.subtilis (duplicates at BM000959360 image!, K000789846 image!, BR0000006600860 image!, P02663388!, P02663405!), but the type is the one [1840] collection cited by Karelin and Kiriloff (1841) distributed as Herb. Fischer no. *504*.

### Poa diaphora subsp. oxyglumis

Taxon classificationPlantaePoalesPoaceae

(Boiss.) Soreng & G.H. Zhu, Fl. China 22: 266. 2006.

Poa persica var. oxyglumis Boiss., Fl. Orient. 5: 610. 1884. Eremopoaoxyglumis (Boiss.) Roshev., Fl. URSS 2: 430, 756, pl. 32, f. 9–10. 1934. Eremopoapersicavar.oxyglumis (Boiss.) Grossh., Fl. Kavkaza (ed. 2) 1: 268. 1939. Eremopoaaltaicasubsp.oxyglumis (Boiss.) Tzvelev, Bot. Zhurn. (Moscow & Leningrad) 51(8): 1104. 1966. Eremopoapersicavar.oxyglumis (Boiss.) Rahmanian, Iran. J. Bot. 21(11): 214. 2014. nom. inval. isonym.

#### Type.

Turkey. In collibus prope Baibout, 17 Jul 1963, *E. Bourgeau* (lectotype, designated by Tzvelev 1976, pg. 479: LE! [LE00009676]; isolectotypes: LE [LE00009678 image!], P [P02358146! pp a, P03142400!]).

#### Distribution.

Armenia, Azerbaijan, Georgia, China (Xizang), Kyrgyz Republic, Pakistan, Tajikistan, Turkey, Turkmenistan and Uzbekistan.

#### Notes.

Most accounts have recognised this taxon at one rank or another, except Mill (1985) who treated it as a synonym of *E.songarica*. Several collections were cited in the original protologue: *Tchihatcheff*, Hab. in Ponto; *Balansa*, Ponto Lazico ad Djimil [*Balansa 1549* G00308631, E, LE!, P02014318 (= subsp. oxyglumis), P02014317 (= P.persicasubsp.multiradiata), US!]; *Huet*, Erzurum [G00330279, G00308633]; *E. Bourgeau*, Armenia, in collibus et agris in cultis Armeniae Turcicae ad Gumuchkhane.

### Poa millii

Taxon classificationPlantaePoalesPoaceae

Soreng, Cabi & L.J. Gillespie nom. nov.

urn:lsid:ipni.org:names:60477374-2

Eremopoa capillaris R.R. Mill, Fl. Turkey & E. Aegean Isl. 9: 624, 490. 1985 (non Poacapillaris L. 1753). Eremopoapersicavar.ramosissima Azn. ex R.R. Mill, Fl. Turkey & E. Aegean Isl. 9: 490. 1985, nom. inval.

#### Type.

Turkey. Adana, distr. Feke, Sencan Dere nr Gurumze, 1300 m, 30 May 1952, *P.H. Davis, Dodds & Cetic 19681* (holotype: E! [E00196495]; isotypes: BM! [BM000959355], K! [K000789852]).

#### Distribution.

Turkey (central and eastern Taurus Mts. and adjacent ranges).

#### Notes.

Morphologically *Poamillii* is intermediate between P.persicasubsp.persica and *P.attalica*. However, we are not sure which of these it is actually related to or if it is a hybrid between them. The type approaches *P.persica* in having anthers 1.2–1.3 mm long and *P.attalica* in having abundant branching and sometimes having some sterile branches amongst the lower branch whorls. Much of the material of *P.millii* from further west than the type location from the Taurus Mts. has smaller anthers and is problematical to separate from *P.attalica*.

### Poa nephelochloides

Taxon classificationPlantaePoalesPoaceae

(Roshev.) Soreng, Cabi & L.J. Gillespie comb. nov.

urn:lsid:ipni.org:names:60477375-2

Eremopoa nephelochloides Roshev., in Köie, M., Beitr. Fl. Sudwest Iran I. Danish Sci. Invest. Iran In K. Jessen & R. Sparck. (Eds) Danish Sci. Invest. Iran, pt. 4: 50. 1945. Eremopoapersicavar.nephelochloides Roshev., nom. inval. as syn. of E.nephelochloides.

#### Type.

Iran. 60 km north of Dizful, 3 May 1937, *M. Köie 475* (lectotype, **here designated**: C [C10016935 image!]; isolectotype: LE).

#### Distribution.

Iran (Zagros Mts.).

#### Notes.

Due to its sterile whorls of branches, this species seems very close to *Poamillii* and *P.attalica*, but may be a derivative of *P.persica* since it has longer anthers than the previous taxa. Roshevits cited two gatherings of *Köie*: “Kechwar, 700 m (3 May 1937; no. 475). Chah-Bazan, 500 m” (Kechvar is about 60 km north of Dizful). The specimen at C has the same date and collection number as Roshevits cited and was annotated by Roshevits as this taxon; we select it as the lectotype. The anthers are ca. 1.1–1.2 mm as measured from the C photo and other characters seem to match *P.attalica*. The anther length is given as 1.5 mm in Roshevits’ diagnosis. The specimen clearly has the hyaline lemma apices of *P.persica* s.l. (in contrast to *P.diaphora*). However, these features are also present in the type of *E.capillaris* (=*P.millii*). *Poaattalica* has shorter anthers, ca. 0.8 to 1 mm, on the type (anthers not described by Scholz 1980 or Mill 1985). *Poanephelochloides* and *P.attalica* may represent the same species, diagnosed as different from *P.persica* by sterile branches and from *Nephelochloaorientalis* Boiss. by glabrous lemmas (*P.nephelochloides* has pubescent lemmas). However, *Poanephelochloides* and *P.attalica* are geographically isolated by over 1500 km and have different anther lengths.

### Poa persica

Taxon classificationPlantaePoalesPoaceae

Trin., Mém. Acad. Imp. Sci. St.-Pétersbourg, Sér. 6, Sci. Math. 1(4): 373. 1830.

Festuca persica (Trin.) K. Koch, Linnaea 21(1[4]): 410. 1848. Nephelochloapersica (Trin.) Griseb., Fl. Ross. 4(13): 366. 1852. Poapamphylica Boiss., Diagn. Pl. Orient., ser. 1, 13: 58. 1854[1853?], nom. inval. as syn. of Poapersica. Eremopoapersica (Trin.) Roshev., Fl. URSS 2: 430, pl. 32, f. 8. 1934.

#### Type.

Iran: in collibus ad Akar-Tschai prob. Karabagh, 1400–1900 m, 27 May 1829, *Szowits 246* (lectotype, designated by Tzvelev 1976, pg. 479: LE! [photo E000327964!, TRIN-microform 434-B4!]; isolectotypes: LE [TRIN-2666.02!, TRIN-microform 434A8!, 434-B1!, 434-B2!, 434-B3!]).

#### Notes.

Other original material includes: Iran, Prov. Aderbeidschan. distr. Khoi, ad Seidchadzi, 18 May 1828, *Szovits 258* (LE!, LE0009678 [image!], LE0009679, LE0009680 [image!], LE0009681 [image!], W0028250 [image!]; In apricis prov. Aderbeidschan e Karabahg, *Fischer* [herb. Fischer] (K000789867 [image!]). *Poapersica* has two major variations: subsp. persica with pubescent lemmas and relatively narrower panicle length to plant height ratio; and subsp. multiflora with glabrous lemmas and relatively greater panicle length to plant height ratio, and often more flowers per spikelet.

### Poa persica subsp. persica

Taxon classificationPlantaePoalesPoaceae

Fig. 3B, C

Eremopoa persica var. typica Grossh., Trudy. Bot. Inst. Azerbaidzh. Fil. Akad. Nauk. S.S.S.R. 8: 268. 1939, nom. inval. Eremopoapersicavar.persica. 1960.
Poa cilicensis Hance, Ann. Sci. Nat., Bot., sér. 4, 18: 234. 1862. Type protologue. In Tauro cilicio, *Kotschy 529*. Type. In monte Tauro, aestate 1836, *Kotschy 529*, this from hb. H.F. Hance [via Reed 1887] no. 7498 (lectotype, **here designated**: BM! [BM000551484, right hand plant (2 left hand specimens are Poadiaphora var. songarica and are clearly excluded from Hance’s description written on the sheet)]; isolectotype: P! [P02642319]).
Glyceria taurica Steud., Syn. Pl. Glumac. 1: 286. 1854 (non Poataurica E. Pojarkova, 1965, Poa×taurica H.N. Pojark., 1963). Type protologue. In monte Tauro, 1836, *Kotschy* (Kotschy hrbr.). Type. In monte Tauro, Aestate,1836, *Kotschy 529* (lectotype, **here designated**: P! [P02642319]; isolectotype: BM [BM000551484 image!]).

#### Distribution.

Armenia, Azerbaijan, Georgia, Egypt (north coast, possibly adventive), Iran, Iraq, Lebanon, Pakistan, Syria, Turkey; waif in France (introduced in wool, Marseille, *H. Roux*, P06768417!, P03370109!; RJS determination, 2015) and Norway (Greuter et al. 1984+).

#### Notes.

Although Kotschy’s herbarium is mainly at W, a search of the W herbarium website did not turn up *Kotschy 529* except as the genus *Arenaria* from Tauro cilicio or a *Scrophularia* from Persia. *Kotschy 528* at W is a *Poa* of the *P.bulbosa* complex from “In monte Tauro” in 1836. Presumably the earlier 1836 set was broken up and *529* ended up at BM and P. The anthers in the *G.taurica* lectotype are 1.8 mm long and the lemmas are pubescent along the keel and marginal veins.

### Poa persica subsp. multiradiata

Taxon classificationPlantaePoalesPoaceae

(Trautv.) Soreng, Cabi & L.J. Gillespie comb. nov.

urn:lsid:ipni.org:names:60477377-2

Fig. 3D, E

Poa palustris var. multiradiata Trautv., Trudy Imp. S.-Peterburgsk. Bot. Sada 4: 406. 1876. Poamultiradiata (Trautv.) Regel, Trudy Imp. S.-Peterburgsk. Bot. Sada 7: 620. 1880. Eremopoamultiradiata (Trautv.) Roshev., Fl. URSS 2: 430, t. 32. 1934. Eremopoapersicasubsp.multiradiata (Trautv.) Tzvelev, Zlaki SSSR 479. 1976.
Nephelochloa tripolitana Boiss. & Blanche, Diagn. Pl. Orient., ser. 2, 4: 133–134. 1859. Poapersicavar.major Boiss., Fl. Orient. 5: 610–611. 1884. Type protologue. Hab. ad margines semitarum inter hortos ad Tripolium Syriae (*Blanche*), circa Byrouth in Libano (*Gaillardot*). Type. Lebanon. S. Tripoli, dans les bords des chemins, 16 May 1854, *Blanche* 1267 (lectotype, **here designated**: JE [JE00005064 ex herb. Gaillardot, image!]). Note. Two of the original specimens turned up in our search, *Blanche 1267* (JE00005064 ex herb. Gaillardot) and *Gaillardot s.n.* (JE00005065 ex herb Gaillardot no. 2323 [image!]). *Blanche* in 1869 (P02530724) might also be original material, with a distribution date rather than a collection date.
Eragrostis barbeyi Post, Bull. Herb. Boissier 5: 760–761. 1897. Type protologue. Habitat in collibus prope Midyat (Mardin), *no. 38*. Type. Turkey. Midyat, Hillsides, May 1895, *38 Barbey* (lectotype, **here designated***by Nada Sinno Saoud & RJS*: BEI! (image seen by RJS!)). Note. The BEI sheet has “No. 55 *38 Barbey*, 1895” (55 was originally written as 54 but the 4 written over by 5).
Eremopoa mardinensis R.R. Mill, Fl. Turkey & E. Aegean Isl. 9: 624, 488. 1985.Type. Turkey. Mardin, Mardin to Nusaybin, 8 km from Mardin, 850 m alt., shallow limestone gully, 22 May 1957, *P. H. Davis & D. Hedge 28491* (holotype: E! [E00196494]).

#### Type.

Armenia rossica, prope monasterium Kiptschach, 1875, *G. Raddi*. Type: Armenia rossica: prope monasterium Kiptschach in monte Alagos, Jun 1875, *G. Radde 124* (holotype: LE! [photo E00326521!]; isotypes: LE, LE, W [W19160014191 image!]).

#### Distribution.

Armenia, Georgia, Iran, Lebanon, Pakistan, Syria and Turkey.

#### Notes.

The presence of hairs on the lemmas in material treated as “*multiradiat*” is confused in the literature. Mill (1985) indicates that *E.multiradiata* and *E.persica* s.s. have lemma keels hairy in the lower ⅓–½. We concur with Tzvelev (1976), who keyed E.persicasubsp.persica as lemmas short pilose along the base of keel and marginal veins and subsp. multiradiata as lemmas glabrous or with a few solitary hairs.

Mill (1985) distinguished his new species *Eremopoamardinensis* from *E.multiradiata* based on its glabrous lemmas, 8–12-flowered spikelets and florets strongly divergent from the rachilla. However, subsp. multiradiata also has glabrous lemmas (as noted above) and divergent florets (when spikelets are in flower) and its (4)5–9(10)-flowered spikelets overlap in number; therefore, we treat *E.mardinensis* as a synonym of *E.multiradiata*. The type material of *Eragrostisbarbeyi* is from the same place as *E.mardinensis* and is clearly the same form (spikelets many-flowered); *Nephelochloatripolitana*, with ca. 12–14-flowered spikelets, also appears to belong to this form. If *E.mardinensis* were accepted as a species, the basionym names *Eragrostisbarbeyi* or *Nephelochloatripolitana* would have priority.

### Poa subg. Pseudopoa sect. Speluncarae

Taxon classificationPlantaePoalesPoaceae

Soreng, Cabi & L.J. Gillespie sect. nov.

urn:lsid:ipni.org:names:60477378-2

#### Type.

*Poaspeluncarum* J.R. Edm.

#### Diagnosis.

Differing from Poasect.Pseudopoa in being perennial and stooling, with top culm sheath margins fused 40–50% their length and from almost all *Poa* in proximal spikelets being 1-flowered.

### Poa speluncarum

Taxon classificationPlantaePoalesPoaceae

J.R. Edm., Fl. Turkey & E. Aegean Isl. 9: 623. 473. 1985.

#### Type.

Turkey. C4, Konya, distr. Ermenek, Kamis Dere between Ermenek and Oyuklu Dag., floor of caverns, 1400–1500 m, 14 Aug 1949, *P. H. Davis 16180* (holotype: K! [K000641325]; isotype: E! [E00367874]).

#### Distribution.

Turkey (central Taurus Mts.).

#### Notes.

*Poaspeluncarum* was described by Edmondson (1985) as an annual species of Poasect.Ochlopoa Asch. & Graebn (≡ Poasect.Micrantherae Stapf. Type: *Poaannua*). Our investigation found it to be a feeble, stooling perennial with sparsely scabrous panicle branches, uppermost sheaths closed up to half their length, spikelets sparsely scaberulous, mostly 1-flowered, the distal-most ones frequently 2(–3) flowered, anthers 1.1–1.7 mm, caryopsis 1.7–1.8 mm long, hilum 0.3 mm long and grain adherent to the palea. DNA data have clearly placed it in the *Poa* clade that includes *Eremopoa* species (E clade), either as sister to *P.attalica* (nuclear data) or as sister to *P.attalica* + *P.sintenisii* (plastid data). The species is odd in subgenus Pseudopoa for its perennial habit (albeit weak) and more closed sheaths, and in *Poa* generally by its mostly uniflorous spikelets. It is a very rare species that lives in the backs of shallow, moist, cool caves in the Taurus Mts., along with other cave endemics.

### Poa subg. Pseudopoa sect. Lindbergella

Taxon classificationPlantaePoalesPoaceae

 (Bor) Soreng, Cabi & L.J. Gillespie sect. nov.

urn:lsid:ipni.org:names:60477379-2

Lindbergia Bor, Svensk Bot. Tidskr. 62: 467, 1968 (nom. illeg. hom., non Kindb., 1897).
Lindbergella Bor, Svensk Bot. Tidskr. 63: 368. 1969.

#### Type.

*Poasintenisii* H. Lindb. ≡ *Lindbergellasintenisii* (H. Lindb.) Bor.

#### Diagnosis.

Differing from Poasect.Pseudopoa in: panicle branches smooth; lower glume 3-veined, up to 3/4 as long as the lower lemma; lemmas 3-veined, relatively firm, sericeous on keel marginal veins and sides; callus with short crown of hairs, the hairs 0.2 mm long; and palea keels sericeous in part.

### Poa sintenisii

Taxon classificationPlantaePoalesPoaceae

H. Lindb., Årsbok-Vuosik. Soc. Sci. Fenn. 20 B (7): 5. 1942 (emend. Lindberg 1946).

Lindbergia sintenisii (H. Lindb.) Bor, Svensk Bot. Tidskr. 62: 467. 1968. Lindbergellasintenisii (H. Lindb.) Bor, Fl. Cyprus 63: 368. 1969.
Poa persica subsp. cypria Sam., Ark. Bot., n.s. 1(9): 417. 1950 [1951].Type. Cyprus. auf dem Troodos, 20 Jun 1880, *P. Sintenis 881* (lectotype, **here designated**: S; isolectotypes: B [B 10 0365891!], LD [LD1808162 image!, LD1808226 image!], G?, K [K000789835 image!, K000789836 image!, K000789837 image!], W [W0012225 image!, W0033518 image!, W00096518 image!, W0019026 image!]).

#### Type protologue.

Cyprus. In pineto (*P.pallasiana*) in m. Troodos lecta est. 1939. **Type.** Cyprus. Troodos in pineto juxta via huad procul ab “Olympus Camp Hotel”, 22 Jun 1939, *H. Lindberg s.n.* (holotype: S [S-11-34137 image!]; isotypes: S [S-G-4941 image!], K [K000789839 image!], LD [LD1807330 image!], W [image!]).

#### Distribution.

Cyprus (Mt. Troodos, endemic to serpentine rocks).

##### Names of uncertain application within *Poa* subgen. *Pseudopoa*

### Festuca bellula

Taxon classificationPlantaePoalesPoaceae

Regel, Trudy Imp. S.-Peterburgsk. Bot. Sada 7: 594. 1881. Eremopoa bellula (Regel) Roshev., Fl. URSS 2: 431, pl. 32, f. 12. 1934.

#### Type protologue.

Ad fontes calidos Araschan Bulak in Turkestania occidentali, *Krause* s.n. **Type**: Taschkenter Alatau, Araschan Bulak, 11 Jun 1871, (*Hieronymous*) *Krause* s.n. (holotype: LE [only one collection cited]).

#### Notes.

*Eremopoabellula* was applied by several authors to small densely tufted alpine annual plants of south-central and southwest Asia, which we recognise as P.diaphoravar.alpina (based on Poapersicavar.alpinaBoissier [1884]). Tzvelev (1976, pg. 480) noted that the holotype collection of *E.bellula* appeared to be a mix of *altaica* (*diaphora*) and *songarica* forms (“p.p. max” = E.altaicasubsp.songarica , somewhat intermediate between this subsp. and subsp. altaica, and “p.p. minor” = E.altaicasubsp.altaica); he considered *E.bellula* to be a synonym of *E.altaica* subsp. songarica. Further study is needed to clarifiy the placement of *Eremopoabellula* and determine if it is synonymous with P.diaphoravar.alpina.

### Eremopoa glareosa

Taxon classificationPlantaePoalesPoaceae

Gamajun., Bot. Mater. Gerb. Inst. Bot. Akad. Nauk Kazahsk. SSR 2: 2. 1964.

#### Type protologue.

Usbekistanica, Tian Schan Occid., Bostandyk, fonts Aksar-sai, 28 Jul 1949, *N. V. Pavlov* s.n. (holotype: AA).

#### Notes.

Tzvelev (1976, pg. 480) included *E.glareosa* as a synonym under E.altaicasubsp.songarica, but noted that it is somewhat intermediate between this taxon and E.altaicasubsp.altaica. As the protologue indicates the plants are 10–28 cm tall, with 3 to 4 florests per spikelet, spikelets 4–7 mm long and anthers 2.5 mm long, this is more likely to be *Poapersica*, perhaps subsp. multiradiata, since no pubescence is indicated.

### Festuca heptantha

Taxon classificationPlantaePoalesPoaceae

K. Koch, Linnaea 21(3): 410. 1848. Poa heptantha (K. Koch) Steud., Syn. Pl. Glumac. 1: 255. 1854.

#### Type protologue.

Im Hochgebirge, auf sumpfigen Wiesen, auf Urgestein, 5500 ft, *C. Koch* s.n. (holotype: B, probably destroyed).

#### Note.

There is no location in the species protologue beyond the article title “Beitrage zu einer Flora des Orients”. Tzvelev (1976) indicated this name and the next, *Festucapolygama*, probably apply to *Eremopoapersica* and that the types of these were in Berlin (B). Clayton et al. 2002+ (GrassBase) reflect the same information. RJS was unable to locate type material of either of these two names at B, P or via internet searches.

### Festuca polygama

Taxon classificationPlantaePoalesPoaceae

K. Koch, Linnaea 21: 409. 1848. Poa polygama (K. Koch) Steud., Syn. Pl. Glumac. 1: 255. 1854.

#### Type protologue.

“Aus dem Wilhelm’schen Herbr als *Poapersica*.” **Type**: *Wilhelms* (holotype: B, probably destroyed).

#### Notes.

Tzvelev (1976) indicates “Caucasus?”, but there is no location in the species protologue beyond the article title “Beitrage zu einer Flora des Orients”.

##### Excluded names

### Eremopoa medica

Taxon classificationPlantaePoalesPoaceae

H. Scholz, Willdenowia 11(1): 96. 1981.

#### Type.

Persia, Prov. Azerbaijan occid.: In pratis paludosis SE Shahpur versus lacum Rezaiyeh (Urmia), 1300 m; 12 Jun 1971, *Rechinger 41820* (holotype: W [W1972-0000975 image!; isotypes: B! [B 10_0272774], GZU [GZU000201751 image!], WU [WU0033125 image!]).

#### Notes.

The type collection of *Eremopoamedica* is clearly a perennial species of *Puccinellia* (possibly *P.gigantea* (Grossh.) Grossh.) with lemmas rounded on the back, a distinct short crown of callus hairs and papillae common on vegetative structures (pedicels and leaves). Material cited as *E.medica* in Rahmanian et al. (2014, fig. 5) appears to us to be Poapersicasubsp.persica; their description and illustration indicate an annual habit, pubescent lemmas and panicles with 10 or more branches per whorl. The single specimen (TARI 35082) cited was included in our molecular analysis and formed a clade with other *P.persica* accessions in all trees.

##### Invalid names, not vouchered

***Festucaamherstiana* Nees, Ill. Bot. Himal. Mts. 417. 1839, nom. nud., name in list, no voucher.**

**Notes.** Kew GrassBase (Clayton et al. 2002+) indicates it is equal to *E.persica*. The specimen K00078950 (ex P) (image!), *Voyage V. Jacquemont aux Indes orient. no. 1902*, has this name on the label. The specimen is certainly *P.diaphora*, not *P.persica*.

## Supplementary Material

XML Treatment for Poa subg. Pseudopoa

XML Treatment for Poa subg. Pseudopoa sect. Pseudopoa

XML Treatment for Poa attalica

XML Treatment for Poa diaphora

XML Treatment for Poa diaphora subsp. diaphora var. diaphora

XML Treatment for Poa diaphora subsp. diaphora var. alpina

XML Treatment for Poa diaphora subsp. diaphora var. songarica

XML Treatment for Poa diaphora subsp. oxyglumis

XML Treatment for Poa millii

XML Treatment for Poa nephelochloides

XML Treatment for Poa persica

XML Treatment for Poa persica subsp. persica

XML Treatment for Poa persica subsp. multiradiata

XML Treatment for Poa subg. Pseudopoa sect. Speluncarae

XML Treatment for Poa speluncarum

XML Treatment for Poa subg. Pseudopoa sect. Lindbergella

XML Treatment for Poa sintenisii

XML Treatment for Festuca bellula

XML Treatment for Eremopoa glareosa

XML Treatment for Festuca heptantha

XML Treatment for Festuca polygama

XML Treatment for Eremopoa medica

## Appendix 1

**Table A1.** *Eremopoa*, *Lindbergella*, *Poa* and outgroup samples used in the phylogenetic analyses. Ingroup samples are arranged by plastid clade (pl), nuclear clade (nr) and section. Voucher information (herbarium indicated in parentheses) and country of origin are provided; where there is no collector or collector number, the herbarium specimen number is given. GenBank Accession numbers are provided for *ITS*, *ETS*, *trnT*-*trnL*-*trnF*, *matK* and *rpoB*-*trnC* sequences for each sample; those in BOLD are new to this study.

pl	nr	Section	Taxon	Voucher	Country	ITS	ETS	TLF	matK	rpoB-trnC
A	A	* Alpinae *	*Poaalpina* L.	*Gillespie 6299* (CAN)	USA, Colorado	GQ324483	GQ324287	DQ353985.2	KM523888	KM524001
A	A	* Alpinae *	*Poabadensis* Haenke ex Willd.	*Hajkova et al. 2004-12* (US)	Bulgaria	GQ324490	GQ324295	GQ324402	KY378861	KY378827
A	A	* Alpinae *	*Poaligulata* Boiss.	(JACA 166095)	Spain	GQ324522	GQ324346	GQ324432.2	KY378876	KY378842
A	A	* Alpinae *	*Poathessala* Boiss. & Orph.	*Gillespie et al. 10400* (CAN)	Turkey	KM523802	KM523729	KM524088	KM523901	KM524014
A	A	* Arenariae *	Poabactrianasubsp.glabriflora (Roshev.) Tzvelev	*Gauba* (IRAN 21237)	Iran	KX118734	KX118716	KX118751	**MH921344**	**MH921369**
A	A	* Arenariae *	*Poabulbosa* L.	*Catalan 13-2000* (UZ)	Spain	EU792388	GQ324297.2	AH015557.3	KY378863	KY378829
A	A	* Arenariae *	Poabulbosasubsp.vivipara (Koeler) Arcang.	*Soreng & Soreng 5814* (US)	USA, Nevada (introd.)	GQ324492	GQ324298	GQ324404	**MH921345**	**MH921370**
A	A	* Arenariae *	Poa sinaica Steud. subsp. sinaica	*Soreng & Cabi 9249* (US)	Turkey	KX118748	KX118731	KX118766	KY378886	KY378852
A	A	* Arenariae *	*Poatimoleontis* Heldr. ex Boiss.	*Soreng et al. 7509-1* (US)	Greece	KX118750	KX118732	KX118768	**MH921354**	**MH921379**
E	E	* Lindbergella *	*Lindbergellasintenisii* (H. Lindb.) Bor	*Hand 6102* (US)	Cyprus	**MH921326**	**MH921310**	**MH921393**	**MH921342**	**MK060117**
E	E	* Pseudopoa *	*Eremopoaattalica* H. Scholz	*Gillespie et al. 10612* (CAN)	Turkey	**MH921313**	**MH921297**	**MH921380**	**MH921329**	**MH921355**
E	E	* Pseudopoa *	*Eremopoamultiradiata* (Trautv.) Roshev.	*Soreng & Cabi 9240* (US)	Turkey	**MH921314**	**MH921298**	**MH921381**	**MH921330**	**MH921356**
E	E	* Pseudopoa *	*Eremopoaoxyglumis* (Boiss.) Roshev.	*Gillespie & Levin 10313* (CAN)	Turkey	**MH921316**	**MH921300**	**MH921383**	**MH921332**	**MH921358**
E	E	* Pseudopoa *	* Eremopoa oxyglumis *	*Gillespie et al. 10578* (CAN)	Turkey	**MH921317**	**MH921301**	**MH921384**	**MH921333**	**MH921359**
E	E	* Pseudopoa *	* Eremopoa oxyglumis *	*Gillespie et al. 10584* (CAN)	Turkey	**MH921318**	**MH921302**	**MH921385**	**MH921334**	**MH921360**
E	E	* Pseudopoa *	* Eremopoa oxyglumis *	*Soreng & Cabi 8855* (US)	Turkey	**MH921315**	**MH921299**	**MH921382**	**MH921331**	**MH921357**
E	E	* Pseudopoa *	*Eremopoapersica* (Trin.) Roshev.	*Assadi & Vosoughi* (TARI 24939)	Iran	**MH921321**	**MH921305**	**MH921388**	**MH921337**	**MH921363**
E	E	* Pseudopoa *	* Eremopoa persica *	*Mozaffarian* (TARI 53671)	Iran	**MH921320**	**MH921304**	**MH921387**	**MH921336**	**MH921362**
E	E	* Pseudopoa *	* Eremopoa persica *	*Soreng & Cabi 9215* (US)	Turkey	KY378812	KY378823	KY378816	KY378879	KY378845
E	E	* Pseudopoa *	* Eremopoa persica *	*Yazdanfard* (IRAN 51968)	Iran	**MH921319**	**MH921303**	**MH921386**	**MH921335**	**MH921361**
E	E	* Pseudopoa *	* Eremopoa persica *	*Mozaffarian & Nowrozi* (TARI 35082)	Iran	**MH921322**	**MH921306**	**MH921389**	**MH921338**	**MH921364**
E	E	* Pseudopoa *	*Eremopoasongarica* (Schrenk ex Fisch. & C.A. Mey.) Roshev.	*Assadi & Mozaffarian* (TARI 36867)	Iran	**MH921324**	**MH921308**	**MH921391**	**MH921340**	**MH921366**
E	E	* Pseudopoa *	* Eremopoa songarica *	*Iranshahr* (IRAN 20357)	Iran	**MH921323**	**MH921307**	**MH921390**	**MH921339**	**MH921365**
E	E	* Pseudopoa *	* Eremopoa songarica *	*Soreng & Güney 4165* (US)	Turkey	EU792400	GQ324311	DQ353988.2	KY378868	KY378834
E	E	* Pseudopoa *	* Eremopoa songarica *	*Soreng & Cabi 9320* (US)	Turkey	**MH921325**	**MH921309**	**MH921392**	**MH921341**	**MH921367**
E	E	* Speluncarae *	*Poaspeluncarum* J.R. Edm.	*Soreng et al. 8202* (US)	Turkey	**MH921328**	**MH921312**	**MH921395**	**MH921353**	**MH921378**
H	P+H	unclassified	*Poapseudobulbosa* Bor	*Soreng et al. 8246* (US)	Turkey	KX118747	KX118729	KX118765	**MH921352**	**MH921377**
H	P+H	* Acutifoliae *	*Poaplanifolia* Kuntze	*Peterson et al. 19233* (US)	Argentina	KM523800	KM523727	KM524087	KM523896	KM524009
H	P+H	* Brizoides *	*Poapoiformis* (Labill.) Druce	*Gillespie et al. 7381* (CAN)	Australia	GQ324534	GQ324361	GQ324445	KM523897	KM524010
H	P+H	* Homalopoa * *s.l.*	*Poareflexa* Vasey & Scribn.	*Soreng 7422* (US)	USA Colorado	GQ324543	KX118730	GQ324450	KY378882	KY378848
H	P+H	* Homalopoa * *s.s.*	*Poaasiae*-*minoris* H. Scholz & Byfield	*Soreng et al. 8100* (US)	Turkey	**MH921327**	**MH921311**	**MH921394**	**MH921343**	**MH921368**
H	P+H	* Homalopoa * *s.s.*	*Poachaixii* Vill.	*Soreng 4677* (US)	Russia	EU792404	GQ324299	EU854590	KM523890	KM524003
H	P+H	* Homalopoa * *s.s.*	* Poa chaixii *	*Soreng 7524* (US)	Germany	GQ324493	GQ324300	GQ324405	**MH921346**	**MH921371**
H	P+H	* Homalopoa * *s.s.*	*Poamasendarana* Freyn & Sint.	*Assadi* (TARI 73254)	Iran	KX118743	KX118725	KX118761	**MH921351**	**MH921376**
H	P+H	* Homalopoa * *s.s.*	*Poaoccidentalis* Vasey	*Peterson & Valdes Rena 18918* (US)	Mexico	KU756540	KU763436	KU763514	KY378877	KY378843
H	P+H	* Homalopoa * *s.s.*	*Poaremota* Forselles	*Soreng et al. 7540* (US)	Kyrgyz Republic	GQ324545	GQ324372	GQ324452	KY378883	KY378849
H	P+H	* Madropoa *	*Poafendleriana* (Steud.) Vasey	*Gillespie 6292* (CAN)	USA, Colorado	EU792403	GQ324319	DQ354027	KY378869	KY378835
H	P+H	unclassified (supersect. Homalopoa)	*Poacalycina* (J. Presl) Kunth	*Peterson et al. 17923* (US)	Peru	EU792425	KU763395	EU792467	KY378864	KY378830
H	P+H	unclassified (supersect. Homalopoa)	*Poamarshallii* Tovar	*Peterson et al. 21546* (US)	Peru	KM523799	KM523726	KM524086	KM523895	KM524008
J	J	* Jubatae *	*Poajubata* A. Kern.	*Soreng et al. 9029-2* (US)	Turkey	KY378810	KY378820	KY378814	KY378873	KY378839
J	J	* Jubatae *	* Poa jubata *	*Soreng et al. 9266* (US)	Turkey	KY378811	KY378821	KY378815	KY378874	KY378840
M	M	* Micrantherae *	*Poainfirma* Kunth	*Catalan 3-2000* (UZ)	Spain	GQ324516	GQ324334	GQ324427	KY378871	KY378837
M	M	* Micrantherae *	*Poasupina* Schrad.	*Soreng & Cayouette 5950-2* (US)	USA, cult. (from Europe)	EU792387	GQ324383	DQ353984	KY378888	KY378854
N	N	* Nanopoa *	*Poatrichophylla* Heldr. & Sart. ex Boiss.	*Soreng et al. 7508* (US)	Greece	GQ324554	GQ324386	GQ324461	KY378889	KY378855
N	N	unclassified	*Poadolosa* Boiss. & Heldr.	*Soreng et al. 7495-1* (US)	Greece	GQ324502	GQ324312	GQ324414	KM523891	KM524004.2
N	N	unclassified	Poaiconiavar.pelasgis (H. Scholz) Soreng	*Gillespie et al. 10492* (CAN)	Turkey	KX118744	KX118726	KX118762	MH898827	MH898844
N	N	unclassified	*Poaursina* Velen.	*Stoneberg SH17* (US)	Bulgaria	GQ324527	GQ324352	GQ324437	KY378892	KY378858
N	S	* Secundae *	*Poacurtifolia* Scribn.	*Soreng & Soreng 6347c-1* (US)	USA, Washington	EU792394	KY378819	DQ353994.2	KY378867	KY378833
N	S	* Secundae *	Poa secunda J. Presl. subsp. secunda	*Soreng & Soreng 5812* (US)	USA, Nevada	EU792393	KU763450	DQ353991	KY378884	KY378850
N	S	* Secundae *	*Poastenantha* Trin.	*Soreng & Soreng 6068-1* (US)	USA, Alaska	KU756554	KU763455	DQ354057.2	KY378887	KY378853
P	P+H	* Macropoa *	*Poadensa* Troitsky	*Soreng & Cabi 9306* (US)	Turkey	KX118738	KX118720	KX118755	**MH921347**	**MH921372**
P	P+H	* Macropoa *	*Poabucharica* Roshev.	*Soreng et al. 7662* (US)	Kyrgyz Republic	KX118735	KX118717	KX118752	KY378862	KY378828
P	P+H	* Macropoa *	*Poadiversifolia* (Boiss. & Balansa) Hack. ex Boiss.	*Gillespie et al. 10529* (CAN)	Turkey	KX118739	KX118721	KX118756	**MH921348**	**MH921373**
P	P+H	* Macropoa *	Poa*iberica* Fisch. & C.A. Mey.	*Soreng et al. 7977* (US)	Russia, Cabardino-Balkaria	KX118741	KX118723	KX118758	**MH921349**	**MH921374**
P	P+H	* Macropoa *	Poa longifolia Trin. subsp. longifolia	*Soreng et al. 7945* (US)	Russia, Cabardino-Balkaria	KX118742	KX118724	KX118760	**MH921350**	**MH921375**
P	P+H	* Macropoa *	Poa sibirica Roshev. subsp. sibirica	*Olonova 2003-45* (CAN)	Russia, Khakasia	GQ324547	KY378824	GQ324455	KY378885	KY378851
P	P+H	* Poa *	*Poairkutica* Roshev.	*Kasanovskiy 2002-7* (CAN)	Russia, Irkutsk	EU792402	GQ324335	DQ354007.2	KY378872	KY378838
P	P+H	* Poa *	Poa pratensis L. subsp. pratensis	*Gillespie et al. 10592* (CAN)	Turkey	KX118746	KX118726	KX118764	KY378880	KY378846
P	X	* Malacanthae *	Poa arctica R. Br. subsp. arctica	*Gillespie & Aiken 5701* (CAN)	Canada, Nunavut	GQ324487	GQ324291	DQ354009	KY378860	KY378826
R	R	* Parodiochloa *	*Poacookii* (Hook.f.) Hook.f.	*Hennion Gen1* (P)	Subantarctic Islands, Crozet I.	EU792383	GQ324306	EU792454	KY378866	KY378832
R	R	* Parodiochloa *	*Poaflabellata* (Lam.) Raspail	*Wright 9NSG* (not vouchered)	South Georgia Islands	EU792381	GQ324321	EU792453	KM523892	KM524005
S	S	* Abbreviatae *	Poa flexuosa Sm. subsp. flexuosa	*Brochmann 2000-3-1* (CAN)	Norway	GQ324520	GQ324342	GQ324418	KY378875	KY378841
S	S	* Abbreviatae *	*Poapseudoabbreviata* Roshev.	*Soreng & Soreng 6032-1* (US)	USA, Alaska	EU792398	GQ324370	DQ353997	KY378881	KY378847
S	S	* Stenopoa *	*Poabiebersteinii* H.N. Pojark. (cf)	*Gillespie & Cabi 10327* (CAN)	Turkey	KY944706	KY944668	KY987089	KY944622	KY987044
S	S	* Stenopoa *	*Poaglauca* Vahl	*Gillespie 5804* (CAN)	Canada, Nunavut	AY237839	GQ324324	GQ324421	KY378870	KY378836
S	S	* Stenopoa *	*Poapalustris* L.	*Gillespie 6461* (CAN)	Canada, Ontario	EU792396	KY378822	DQ354000	KY378878	KY378844
S	S	* Tichopoa *	*Poacompressa* L.	*Gillespie 6457* (CAN)	Canada, Quebec	EU792395	KY378818	DQ354003	KY378865	KY378831
V	V	* Pandemos *	Poa trivialis L. subsp. trivialis	*Soreng 4681-1* (US)	USA, Maryland (introd.)	GQ324555	GQ324387	GQ324462	KY378891	KY378857
V	V	* Pandemos *	Poatrivialissubsp.sylvicola (Guss.) H. Lindb.	*Gillespie et al. 10368* (CAN)	Turkey	KY378813	KY378825	KY378817	KY378890	KY378856
Y	Y	* Sylvestres *	*Poaautumnalis* Elliott	*Soreng 4680* (US)	USA, Maryland	EU792379	GQ324294	DQ353979	KM523889	KM524002
Y	Y	* Sylvestres *	*Poasaltuensis* Fernald & Wiegand	*Gillespie 7043* (CAN)	Canada, Ontario	EU792378	GQ324374	EU792451	KM523899	KM524012
Y	Y	* Sylvestres *	*Poawolfii* Scribn.	*Soreng & Soreng 5800* (US)	USA, Missouri	EU792377	GQ324389.2	AH015556.2	KY378893	KY378859
		outgroup	*Arctagrostislatifolia* (R. Br.) Griseb.	*Gillespie et al. 6586* (CAN)	Canada, Nunavut	EU792351	GQ324245	DQ353969	KM523924	KM523954
		outgroup	*Miliumeffusum* L.	*Soreng 7771* (US)	Sweden	KM523785	KM523711	KM524072	KM523870	KM523983
		outgroup	*Nicoraepoaandina* (Trin.) Soreng & L.J. Gillespie	*Soreng & Soreng 7182* (US)	Chile	EU792354	GQ324275	DQ353971	KM523874	KM523987
		outgroup	*Phleummontanum* K. Koch	*Gillespie et al. 10614-2* (CAN)	Turkey	KM523793	KM523720	KM524081	KM523883	KM523996
		outgroup	Phleum pratense L.	*Soreng 7943* (US)	Russia, Stavropol	KM523796	KM523723	KM524084	KM523886	KM523999

## Supplementary materials

Supplementary material 1 Table S1. Characteristics of the DNA alignments and data partitions and parameters and summary statistics of the PAUP and Bayesian analyses Data type: (measurement/occurence/multimedia/etc.) Explanation note: Five DNA sequence alignments for *Poa* were analysed: ETS, ITS, *matK, rpoB-trnC* and *trnT-trnL-trnF* (TLF). For each data partition (five individual markers, plastid, nuclear and combined), the number of samples and the total number of aligned characters are given. For the PAUP analyses, the following statistics are given: the number of parsimony informative (PI) characters, percentage of characters that are parsimony informative, maximum parsimony (MP) tree length (L), number of most parsimonious trees, consistency index excluding uninformative characters (CI) and retention index (RI). Parameters used and statistics of the Bayesian analyses, as determined by the Akaike Information Criterion (AIC) implemented in jModeltest, are given as follows: likelihood score (-InL), number of substitution schemes, substitution rates (rAC, rAG, rAT, rCG, rCT, rGT), character state frequencies (fA, fC, fG, fT), substitution model, proportion of invariable sites and gamma shape parameter. This is an open access article distributed under the terms of the CC0 Public Domain Dedication.Lynn J. Gillespie, Robert John Soreng, Evren Cabi, Neda Amiri
